# PANDAS Syndrome: A Narrative Review of the Diagnostic Conundrum in Children with Acute Neuropsychiatric Symptoms

**DOI:** 10.3390/ijms27104612

**Published:** 2026-05-21

**Authors:** Carlo Alberto Cesaroni, Giulia Pisanò, Susanna Rizzi, Agnese Pantani, Daniele Frattini, Carlo Fusco

**Affiliations:** 1 Child Neurology and Psychiatry Unit, Mother and Child Department Santa Maria Nuova, AUSL-IRCCS di Reggio Emilia, 42123 Reggio Emilia, Italy; carloalberto.cesaroni@ausl.re.it (C.A.C.); susanna.rizzi@ausl.re.it (S.R.); agnese.pantani@ausl.re.it (A.P.); daniele.frattini@ausl.re.it (D.F.);; 2 Pediatric Neurophysiology Laboratory, Mother and Child Department, Azienda USL-IRCCS di Reggio Emilia, 42123 Reggio Emilia, Italy

**Keywords:** PANDAS, PANS, paediatric movement disorders, anti-streptolysin O, dopamine receptor autoantibodies, chorea, tic disorder, Tourette syndrome, heritability, Sydenham chorea, autoimmune encephalitis

## Abstract

The hypothesis that Group A beta-haemolytic Streptococcus (GAS) triggers an autoimmune cascade targeting basal ganglia dopaminergic circuits—producing obsessive–compulsive disorder (OCD), tic disorders, or chorea depending on the receptor subtype involved—is biologically compelling and supported by emerging molecular evidence. Yet PANDAS has remained a diagnostic conundrum since its original description in 1998, with ongoing uncertainty surrounding diagnostic criteria, the interpretation of streptococcal serology, and the distinction from primary neurodevelopmental disorders. This study aimed to review the diagnostic challenges of PANDAS, with focus on streptococcal serology interpretation, advances in dopamine receptor autoantibody biology, the genetic epidemiology of primary tic disorders, and the differential diagnosis of acute neuropsychiatric presentations in children. A structured narrative review was conducted using PubMed, MEDLINE, EMBASE, and the Cochrane Library for publications from 1998 to early 2025 addressing PANDAS, PANS, streptococcal antibodies, childhood movement disorders, autoimmune encephalitis, and the genetics of tic disorders. No currently available biomarker—including ASO, anti-DNase B, anti-basal-ganglia antibodies, or the Cunningham Panel—has demonstrated adequate individual-level diagnostic accuracy for PANDAS. Emerging molecular evidence identifies anti-D1R autoantibodies, acting via G protein-and beta-arrestin-mediated signalling, as candidate biomarkers for PANDAS/PANS neuropsychiatric phenotypes, and anti-D2R autoantibodies for Sydenham chorea movement phenotypes; independent replication in unselected populations is required. Primary tic disorders carry heritability estimates of 50–80% and first-degree familial risk ratios of approximately 18-fold in large population-based cohorts. Prospective blinded studies have not demonstrated a consistent population-level association between GAS infections and tic or OCD exacerbations: PANDAS and PANS remain diagnoses of exclusion. The high background prevalence of both GAS exposure and primary neurodevelopmental disorders in overlapping paediatric age ranges creates conditions for incidental temporal co-occurrence. In the absence of validated molecular biomarkers, diagnostic imprecision carries direct clinical consequences: children may be exposed to treatments with significant risk profiles—including IVIG, plasma exchange, and prolonged antibiotic prophylaxis—while evidence-based therapies are delayed. A stepwise diagnostic approach incorporating the full differential diagnosis is both an epistemological and a patient safety imperative.

## 1. Introduction

The relationship between infection and neuropsychiatric dysfunction in children has been recognised for more than a century. Sydenham chorea (SC), the neurological manifestation of acute rheumatic fever (ARF) caused by Group A beta-haemolytic Streptococcus (GAS), provided early clinical and experimental evidence that post-infectious immune mechanisms can produce movement and behavioural disorders in childhood [1,2]. Subsequent work on cross-reactive anti-neuronal antibodies contributed to the hypothesis that a broader spectrum of neuropsychiatric symptoms might arise through related mechanisms [3,4]. In 1998, Swedo and colleagues proposed the Pediatric Autoimmune Neuropsychiatric Disorders Associated with Streptococcal infections (PANDAS) construct, describing children with obsessive–compulsive disorder (OCD) and tic disorders characterised by prepubertal onset, abrupt exacerbations, and temporal association with GAS infection [5]. Five clinical criteria were specified: (i) OCD and/or a tic disorder, (ii) prepubertal onset, (iii) an episodic course, (iv) temporal relationship with GAS infection, and (v) neurological abnormalities during exacerbations [5]. This description was explicitly presented as a provisional research framework rather than a validated clinical diagnosis [5,6]. Over subsequent decades, PANDAS accumulated both supportive and contradictory evidence. Studies reported anti-neuronal reactivity, basal ganglia volumetric changes, and immunomodulatory responses in selected cohorts; conversely, prospective blinded investigations did not confirm a consistent population-level association between GAS infections and neuropsychiatric exacerbations [6,7,8,9,10,11,12,13]. The broader concept of Pediatric Acute-onset Neuropsychiatric Syndrome (PANS) was subsequently proposed to encompass abrupt-onset presentations triggered by varied infectious and non-infectious factors [14]. PANDAS is therefore best understood as a GAS-specific subset within the broader PANS spectrum; while both share the defining feature of abrupt neuropsychiatric onset, PANS does not require streptococcal documentation and encompasses a wider range of putative triggers [14,15]. In 2025, the American Academy of Pediatrics (AAP) published its first clinical report on PANS, characterising it as a likely valid syndrome while specifying the absence of disease-specific biomarkers and recommending caution regarding invasive immunotherapies [16]. This narrative review examines the evidence base relevant to the clinical question of how acute neuropsychiatric symptoms and elevated streptococcal serology should be interpreted together in a child, with reference to the differential diagnosis, available biomarkers, and the genetic epidemiology of primary tic disorders.

## 2. Methods

This narrative review was conducted through a structured search of PubMed, MEDLINE, EMBASE, and the Cochrane Library, covering publications from 1998—the year of the original PANDAS description—to early 2025. Search terms included: PANDAS, PANS, pediatric acute-onset neuropsychiatric syndrome, streptococcal antibodies, anti-streptolysin O, dopamine receptor autoantibodies, childhood movement disorders, Sydenham chorea, autoimmune encephalitis, tic disorders and Tourette syndrome genetics. Reference lists of retrieved articles were hand-searched for additional relevant sources. Inclusion was based on relevance to the clinical and scientific questions addressed: diagnostic criteria, serological and molecular biomarkers, differential diagnosis, genetic epidemiology of primary tic disorders, pathophysiological mechanisms and therapeutic evidence. No formal eligibility criteria or PRISMA flowchart was applied, consistent with the narrative review methodology. Article selection and inclusion decisions were made by the authors through consensus.

## 3. Results

The search yielded a broad literature base spanning original research articles, prospective cohort studies, population-based epidemiological studies, systematic reviews, case series, and clinical guidance documents. Key evidence streams identified included: prospective blinded studies on the association between GAS infection and neuropsychiatric exacerbations; molecular studies on dopamine receptor autoantibody biology; population-based genetic epidemiology of tic disorders; independent evaluations of commercially available biomarker panels; and the 2025 AAP clinical report on PANS. The body of evidence retrieved informed the thematic structure of this review, as detailed in the sections below.

### 3.1. Case Definition and Diagnostic Criteria

The original PANDAS criteria were formulated as a research construct to facilitate case ascertainment, not as a validated clinical instrument [5,6]. Research criteria are intentionally inclusive; the direct translation of research case definitions into routine clinical practice is recognised across medicine as a source of diagnostic imprecision [6,7,8]. Kurlan and Kaplan reviewed the available evidence and concluded that, until more definitive proof is forthcoming, there is an insufficient basis to support routine serological testing for GAS in children presenting with neuropsychiatric symptoms, or the clinical use of antibiotic or immunological treatments [15]. Several elements of the PANDAS criteria are difficult to operationalise in clinical practice. Abrupt onset and episodic course are not quantitatively defined; retrospective reconstruction of symptom timelines is subject to recall bias. The 2013 PANS Consensus Conference proposed onset occurring over 24–48 h as a working threshold [17], though this definition was not derived from systematic empirical data. In practice, the distinction between truly explosive symptom onset and the waxing–waning natural history of primary tic disorders and OCD can be particularly difficult to establish retrospectively [6,15,18]. Gilbert, Mink and Singer proposed a clinically useful stratification of children with fulminant-onset neuropsychiatric presentations into two groups based on the presence or absence of concurrent neurological signs: children with sudden-onset psychiatric symptoms accompanied by neurological findings—including a new non-tic movement disorder, seizures, fluctuations in arousal, or autonomic instability—warrant extensive diagnostic evaluation including neuroimaging and cerebrospinal fluid (CSF) studies, whereas children with psychiatric symptoms alone should first receive evidence-based psychiatric treatment before pursuing invasive investigations [19]. The temporal association with GAS infection is theoretically the most specific criterion, but its discriminative value is limited by the high background prevalence of GAS pharyngitis in school-age children, which creates the conditions for incidental temporal co-occurrence [20,21]. The 2025 AAP clinical report confirmed that PANS is likely a valid syndrome but specified that children with OCD or tics should not routinely receive a PANS workup unless symptom onset is extremely abrupt, and that no specific antibodies or infections have been definitively identified as causative [16]. Both PANS and PANDAS are classified as diagnoses of exclusion in current guidance [16,17].

### 3.2. Laboratory Investigations: Evidence and Limitations

#### 3.2.1. Anti-Streptolysin O and Anti-DNase B Titres

ASO and anti-DNase B titres document prior GAS exposure and do not independently establish causation in neuropsychiatric presentations [22]. Kaplan and colleagues established age-related upper limits of normal in healthy US children, documenting wide variation by age and season [23]. Danchin and colleagues reported analogous data in Australian children [24]. Interpretation of streptococcal serology requires awareness of several sources of potential error: ASO rises from approximately one week after GAS infection, peaks at 3–5 weeks, and begins to decline by 8 weeks; anti-DNase B rises more slowly, reaching maximum levels at 6–8 weeks, and declines over a longer time course, conferring greater utility in delayed presentations such as late-presenting Sydenham chorea [23,24]. Interpretation is further complicated by substantial age- and season-related variation in titres among healthy school-age children—precisely the population at peak PANDAS risk—and by the requirement for locally validated reference ranges [23,24]. Anti-DNase B titres remain elevated for longer periods after GAS infection and may retain utility in delayed presentations such as late-presenting Sydenham chorea [23,24]. Prospective blinded investigations by Kurlan et al. [9] and Leckman et al. [10] did not demonstrate a consistent association between documented GAS infections and subsequent tic or OCD exacerbations. Serial measurement of immune markers across clinical exacerbations does not strengthen the evidence for a causal relationship: Singer and colleagues found no correlation between serial serum immune marker levels and clinical exacerbations in a cohort of children meeting PANDAS criteria, raising fundamental concerns about the viability of autoimmunity as the operative pathophysiological mechanism in this population [25].

#### 3.2.2. Anti-Neuronal and Anti-Basal-Ganglia Antibodies

Kirvan and colleagues demonstrated that SC patient antibodies bind lysoganglioside and dopamine receptor epitopes and activate calmodulin-dependent protein kinase II (CaMKII) signalling [3], with subsequent work identifying beta-tubulin as an additional cross-reactive neuronal target [4]. Dale and colleagues described dyskinesias and neuropsychiatric symptoms following streptococcal infections, consistent with an immune-mediated basal ganglia pathophysiology in susceptible individuals [26]. A mechanistically significant advance concerns dopamine receptor autoantibodies. Ben-Pazi and colleagues demonstrated that the anti-D2R/anti-D1R autoantibody ratio correlates with neuropsychiatric symptom severity in SC [27]. Menendez and colleagues extended these observations across four independent cohorts totalling over 900 patients: anti-D1R autoantibodies were selectively elevated in PANDAS/PANS patients with neuropsychiatric features (tics, OCD), while anti-D2R autoantibodies predominated in Sydenham chorea with choreiform movements [28]. Mechanistically, D1R-reactive patient autoantibodies activate both G protein- and beta-arrestin-transduced D1R signals and sensitise the receptor to dopamine, providing a molecular correlate for the OCD/tic phenotype [28]. It is important to note, however, that anti-D2R positivity has been reported in a proportion of control subjects as well as patients; it is the titre level and the anti-D1R/anti-D2R ratio, rather than binary positivity, that carry phenotypic discriminatory value [27,28]. These findings represent an important advance in the understanding of dopaminergic autoimmunity in this context, although independent replication in unselected population-based cohorts is required before clinical implementation. Chain and colleagues found anti-D1R and anti-D2R antibodies in serum and CSF of PANDAS and SC patients, with CSF positivity in 91% of one PANDAS cohort [29]. Xu and colleagues demonstrated elevated IgG binding to striatal cholinergic interneurons (CINs) in PANDAS patients, normalising after intravenous immunoglobulin (IVIG), identifying a potential cellular mechanism for symptom generation [30]. Earlier studies found that anti-basal-ganglia antibody testing did not reliably distinguish PANDAS patients from controls [11]. The commercially available Cunningham Panel—which comprises a CaMKII neuronal cell activation assay and anti-D1R, anti-D2R, anti-lysoganglioside and anti-tubulin antibody titres—has been evaluated in one independent blinded study by Hesselmark and Bejerot [12]: sensitivities of 15–60% and positive predictive values of 17–40% were reported for individual biomarkers, with 86% of healthy controls meeting at least one positivity criterion compared to 92% of suspected PANS/PANDAS patients; subsequent analysis by the same group further demonstrated no significant difference in CaMKII activation between confirmed PANS patients and healthy controls, with healthy controls showing higher anti-lysoganglioside and anti-β-tubulin values than confirmed PANS cases [31]. A manufacturer-affiliated evaluation demonstrated correlation between Cunningham Panel changes and symptom changes pre/post-treatment in already-diagnosed patients [32], but this design does not assess diagnostic accuracy in unselected populations. The Cunningham Panel is not endorsed in current PANS guidance [16], and its clinical use as a diagnostic instrument requires further independent validation.

#### 3.2.3. Neuroimaging

Early magnetic resonance imaging (MRI) studies reported basal ganglia enlargement in PANDAS [13], but this finding has not been consistently replicated and has also been reported in Tourette syndrome [18,33]. Neuroimaging contributes primarily to the exclusion of structural, demyelinating, or inflammatory disease rather than to confirmation of PANDAS. Electroencephalogram (EEG), CSF analysis, and targeted neuronal antibody panels are appropriate when autoimmune encephalitis is considered in the differential diagnosis [34,35].

### 3.3. Hyperkinetic Movement Disorders in Childhood: The Differential Diagnosis

A hyperkinetic movement disorder in a child is a clinical sign that requires systematic evaluation. Table 1 summarises the principal differential diagnosis relevant to PANDAS and related presentations.

**Figure 1:**
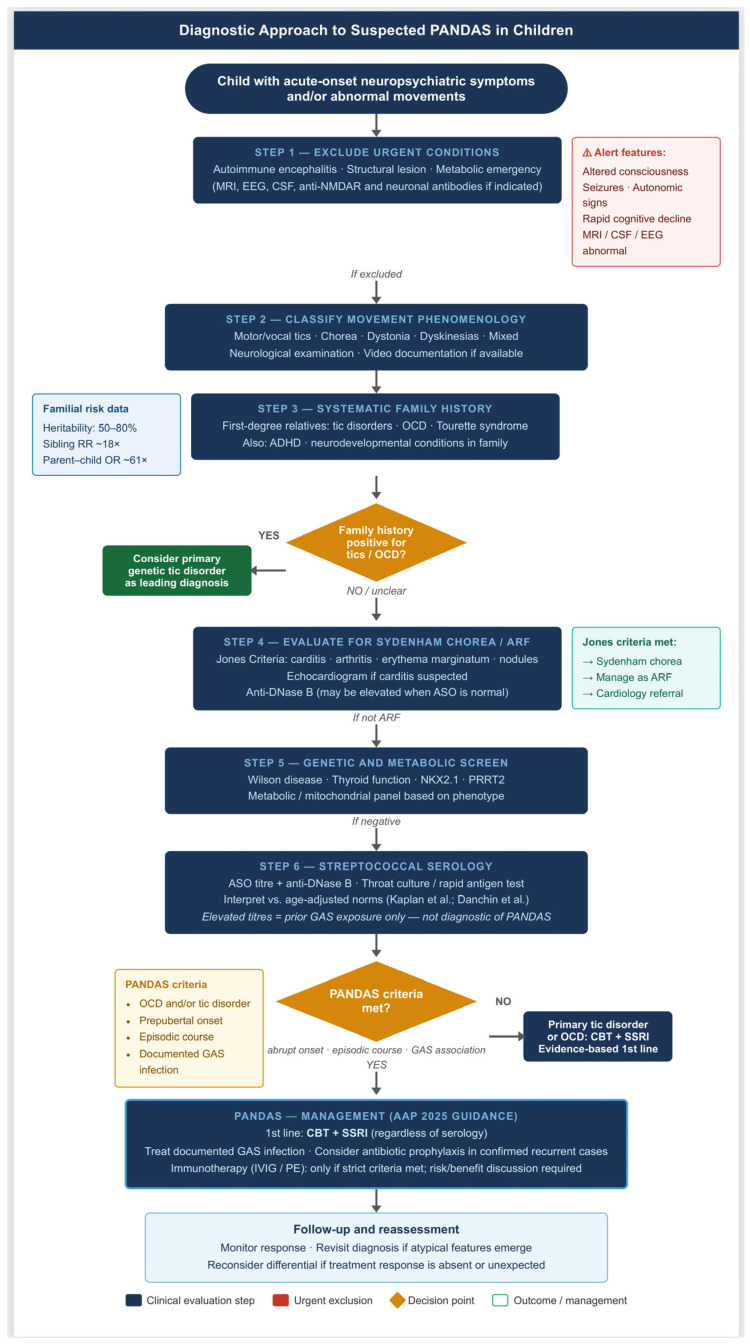
Stepwise diagnostic algorithm for the evaluation of children presenting with acute neuropsychiatric symptoms and/or abnormal movements. The algorithm proceeds through six sequential steps: (1) exclusion of urgent conditions including autoimmune encephalitis, structural lesion, and metabolic emergency; (2) classification of movement phenomenology; (3) systematic family history for tic disorders, OCD, and related neurodevelopmental conditions—a positive family history raises pre-test probability for a primary heritable tic disorder as the leading diagnosis; (4) evaluation for Sydenham chorea and acute rheumatic fever per Jones Criteria; (5) genetic and metabolic screening; and (6) streptococcal serology. Elevated ASO or anti-DNase B titres document prior GAS exposure but are not individually diagnostic of PANDAS. PANDAS and PANS remain diagnoses of exclusion; no currently available biomarker—including the Cunningham Panel—has demonstrated adequate individual-level diagnostic specificity. First-line management for both PANDAS and primary tic disorders/OCD is cognitive behavioural therapy (CBT) combined with selective serotonin reuptake inhibitor (SSRI) pharmacotherapy, regardless of serology. Immunotherapy (IVIG, plasma exchange) should be considered only when strict diagnostic criteria are met and after formal risk/benefit discussion. ARF = acute rheumatic fever; ASO = anti-streptolysin O; CBT = cognitive behavioural therapy; GAS = Group A Streptococcus; IVIG = intravenous immunoglobulin; OCD = obsessive–compulsive disorder; PANDAS = Pediatric Autoimmune Neuropsychiatric Disorders Associated with Streptococcal infections; PE = plasma exchange; TS = Tourette syndrome. Based on [5,16,17,23,24].

A stepwise diagnostic algorithm for the evaluation of children with acute neuropsychiatric symptoms and/or abnormal movements is presented in Figure 1.

#### 3.3.1. Primary Tic Disorders, Tourette Syndrome, and Familial Risk

Tourette syndrome (TS) and other primary tic disorders are the most common causes of chronic motor hyperkinesis in school-age children, with TS prevalence estimates ranging from approximately 0.3% to 1.0% and higher rates for transient tic forms [18]. The natural history of tic disorders is characterised by a waxing–waning course modulated by stress, fatigue, and intercurrent illness. Family-based and population-based genetic studies have documented substantial familial aggregation of tic disorders. Twin studies yield heritability estimates of 50–80% [36]. In a multigenerational population cohort of over 4800 individuals with TS or chronic tic disorders from the Swedish National Patient Register, Mataix-Cols and colleagues found first-degree familial risk approximately 18-fold higher than the general population (OR 18.69; 95% CI 14.53–24.05), declining proportionally with genetic distance [37]. In a Danish national birth cohort of 1.7 million individuals, Browne and colleagues reported a sibling recurrence risk ratio of 18.63 (95% CI 15.34–22.63) and a parent-to-offspring risk ratio of 61.02 (95% CI 44.43–83.82) for TS/chronic tic disorder [38]. Brander and colleagues demonstrated that tic-related OCD clusters more strongly in families than non-tic-related OCD (sibling hazard ratio 10.63 vs. 4.52), indicating a shared familial diathesis [39]. These epidemiological data are relevant to the assessment of children presenting with acute tics or OCD and elevated streptococcal serology. A systematic family history for tic disorders, OCD, and related neurodevelopmental conditions is an important component of the clinical evaluation and contributes to the pre-test probability estimation for both primary genetic tic disorders and PANDAS.

#### 3.3.2. Sydenham Chorea and Acute Rheumatic Fever

Sydenham chorea must be distinguished from PANDAS. It presents as a major manifestation of ARF within the Jones criteria, typically with a more severe generalised choreiform syndrome [2,40,41]. The presence of carditis, arthritis, erythema marginatum, or subcutaneous nodules supports this diagnosis [40]. Rarely, SC may present in a paralytic form with hypotonia and profound weakness in addition to or instead of overt choreiform movements; in such cases, corticosteroid treatment has been reported to be beneficial [42]. The molecular distinction—anti-D2R predominance in SC versus anti-D1R predominance in PANDAS—may provide a useful phenotypic differentiator as these biomarkers undergo further validation [27,28]. ASO titres may be within normal limits when neurological symptoms appear several weeks after the inciting infection; anti-DNase B measurement is therefore recommended in this context [2,41].

#### 3.3.3. Autoimmune Encephalitis

Anti-NMDAR encephalitis and related conditions may present with movement disorders—orofacial dyskinesias, choreoathetosis, dystonia, and stereotypies—in combination with psychiatric symptoms, seizures, cognitive decline, autonomic instability, or altered consciousness [35,43,44]. Prompt recognition is clinically important given the potential for long-term sequelae if treatment is delayed. Evaluation including MRI, EEG, CSF analysis, and targeted neuronal antibody testing is indicated when autoimmune encephalitis is in the differential diagnosis [34,35].

#### 3.3.4. Genetic, Metabolic, and Structural Aetiologies

The differential diagnosis further includes Wilson disease, NKX2.1 mutations (benign hereditary chorea), PRRT2-related paroxysmal dyskinesias, juvenile Huntington disease, metabolic and mitochondrial disorders, drug-induced movement disorders, thyroid disease, and functional neurological disorder [45,46]. Several of these genetic conditions merit particular attention in the context of the PANDAS differential diagnosis, as their hyperkinetic phenotype can be episodic or fluctuating and may be misattributed to a post-infectious mechanism in the absence of genetic testing. ADCY5 mutations produce a distinctive movement disorder characterised by paroxysmal myoclonic jerks, chorea, and dystonia with a variable and often fluctuating course that may superficially resemble an immune-mediated presentation [47]. PRRT2 mutations cause paroxysmal kinesigenic dyskinesias and, as reported in a familial case, may present with phenotypic constellations that extend beyond classical paroxysmal choreoathetosis to include infantile convulsions [48]. NKX2.1 (TITF1) mutations cause benign hereditary chorea, a non-progressive genetic condition that may closely mimic the choreiform presentation seen in post-infectious or immune-mediated disorders; its recognition as a distinct monogenic entity is essential to avoid diagnostic misattribution [49]. Genetic evaluation—by targeted panel or exome sequencing depending on phenotype—is indicated when the movement disorder shows features atypical for primary tic disorder: choreiform or dystonic components, paroxysmal pattern, progressive course, family history of a movement disorder not consistent with TS or OCD, or when a reliable temporal association with GAS infection cannot be established [19,45,46,47,48,49]. Elevated streptococcal antibodies require interpretation within this broader clinical context.

### 3.4. Epidemiological Considerations

GAS pharyngitis is among the most common bacterial infections of childhood [1,21]; TS affects approximately 1% of school-age children with higher prevalence for transient tic forms [18]; and childhood OCD has a prevalence of approximately 1–3% [50]. Because GAS infection, tic disorders, and OCD all occur commonly in overlapping paediatric age ranges, temporal co-occurrence is expected on probabilistic grounds alone. Prospective blinded studies have addressed this overlap. Kurlan and colleagues found no significant association between documented GAS infections and subsequent tic or OCD exacerbations in a prospective cohort [9]. Perrin and colleagues found no increased short-term neuropsychiatric risk in children with documented GAS infection compared to viral-illness controls [51]. Leckman and colleagues reached comparable conclusions in a longitudinal study [10]. These population-level data do not exclude the existence of a susceptible biological subgroup, but they indicate that elevated streptococcal titres are not sufficient, at the population level, to predict neuropsychiatric exacerbations [9,10,51,52]. The available literature derives predominantly from referral cohorts in high-income settings. Gabbay and colleagues explicitly noted that their specialty clinic cohort was largely white and middle-class—a finding attributed to the self-payment requirement of their centre—and cautioned that this demographic distribution may not reflect the broader metropolitan population [53]. Ascertainment bias reflecting differential access to specialist evaluation is therefore a plausible explanation for apparent demographic clustering in PANDAS diagnosis rates, rather than true variation in biological susceptibility, and represents a recognised methodological limitation of the existing evidence base [15,19,53]. Whether biological susceptibility varies across ethnic or socioeconomic groups cannot be determined from current data, and this constitutes an explicit research gap.

### 3.5. Pathophysiological Mechanisms and Animal Models

The PANDAS hypothesis is grounded in molecular mimicry between streptococcal epitopes and basal ganglia neuronal antigens [3]. This mechanism is established for Sydenham chorea [2,3,40]. Kirvan and colleagues demonstrated binding of SC patient antibodies to lysoganglioside and dopamine receptor epitopes with CaMKII activation [3], and subsequently identified beta-tubulin as a cross-reactive target [4]. The most significant recent mechanistic advance is the molecular characterisation of dopamine receptor autoantibody biology. Menendez and colleagues, in a multi-cohort study across four independent groups of over 900 patients, demonstrated that anti-D1R autoantibodies—through G protein- and beta-arrestin-mediated signalling—sensitise D1R to dopamine and are selectively elevated in PANDAS/PANS neuropsychiatric phenotypes; anti-D2R antibodies, which inhibit cAMP and increase dopamine release, predominate in SC with choreiform movements [28]. Ben-Pazi and colleagues had previously shown that the anti-D2R/anti-D1R ratio correlates with symptom severity in SC [27]. This mechanistic dichotomy provides a coherent molecular framework for the clinical distinction between PANDAS and SC, pending independent population-based replication. Chain and colleagues documented anti-D1R and anti-D2R antibodies in serum and CSF of PANDAS and SC patients, with high CSF positivity in one PANDAS cohort [29]. Xu and colleagues demonstrated elevated IgG binding to striatal CINs in PANDAS patients normalising after IVIG, providing a candidate cellular mechanism for tic-like and OCD-like behaviours [30]. Brimberg and colleagues developed a Lewis rat model in which GAS antigen immunisation produced motor and compulsive-like behaviours reversed by haloperidol and paroxetine, with antibody deposition in the striatum, thalamus, and frontal cortex; passive IgG transfer reproduced the phenotype in naïve recipients [54]. Whether only a genetically susceptible subgroup of children develops post-streptococcal neuropsychiatric pathology is a clinically important unresolved question. Early work identified the B-cell alloantigen D8/17—a trait marker of rheumatic fever susceptibility—at significantly elevated frequencies in children with PANDAS (85%) and Sydenham chorea (89%) compared with healthy controls (17%), suggesting a shared immunogenetic diathesis with acute rheumatic fever [55]. The inheritance pattern of D8/17 positivity appears consistent with autosomal recessive transmission, independent of the HLA system [55]. A broader familial autoimmune predisposition has also been described: Murphy and colleagues documented a higher-than-expected rate of autoimmune disease—particularly among mothers—in families of children presenting with tics and/or OCD, consistent with a heritable immune dysregulation background in this population [56]. These observations suggest that individual susceptibility to post-streptococcal neuropsychiatric sequelae may be determined by a combination of immunogenetic factors that remain incompletely characterised. Whether these mechanisms operate in all individuals meeting PANDAS criteria or in a susceptible subset remains an unresolved question.

## 4. Discussion

### 4.1. Clinical and Therapeutic Considerations

A systematic family history for tic disorders, OCD, and related neurodevelopmental conditions is an important clinical element in the evaluation of children presenting with acute neuropsychiatric symptoms. Mataix-Cols et al. [37] and Browne et al. [38] have documented the substantial familial aggregation of tic disorders in large population-based cohorts, data which are relevant to pre-test probability estimation in this context. The AAP 2025 clinical report specifies that children with OCD or tics should not routinely undergo a PANS workup unless symptom onset is extremely abrupt, and that CBT and SSRIs remain evidence-based first-line treatments regardless of presumed aetiology [16]. It is important to emphasise that the presence of a tic disorder alone—even when temporally associated with a streptococcal infection—is insufficient to establish a diagnosis of PANDAS or PANS. Tics are among the most common neurological symptoms of childhood, with a prevalence substantially exceeding that of post-streptococcal neuropsychiatric disease, and their waxing–waning natural history creates the conditions for apparent but coincidental temporal associations with intercurrent infections [9,10,18]. A PANDAS diagnosis requires not only tics or OCD, but also an extremely abrupt onset, an episodic course, and a documented temporal relationship with GAS infection, in the absence of an alternative explanation [5,16,17]. Children with pre-existing neurodevelopmental conditions—including autism spectrum disorder (ASD), subthreshold OCD and anxiety disorders—present a particular diagnostic challenge. The waxing–waning course of behavioural symptoms in children with ASD, and the non-specific exacerbation of these symptoms during intercurrent illness, can be mistaken for the episodic pattern described in PANDAS. The high baseline prevalence of neurodevelopmental conditions in the school-age population further increases the probability of incidental temporal co-occurrence with GAS exposure. Current evidence does not identify ASD as an independent risk factor for PANDAS, and a PANDAS attribution in a child with ASD should be made only after rigorous exclusion of symptom fluctuation attributable to the underlying neurodevelopmental condition [16,19].

A related clinical consideration concerns premorbid neurodevelopmental traits. The PANDAS criteria require symptom onset from a stable premorbid baseline [5,17]; however, subclinical OCD traits, separation anxiety, and behavioural rigidity are common in school-age children and may not be recognised or reported by families until a dramatic exacerbation brings them to clinical attention. In this context, what appears to be an abrupt de novo onset may in some cases represent an acute exacerbation of pre-existing, previously unrecognised vulnerability. Murphy and colleagues found that family rates of OCD among first-degree relatives of PANDAS probands were substantially elevated (26%), consistent with a shared heritable neurodevelopmental diathesis that predates any infectious exposure [56]. These neurodevelopmental and immunogenetic susceptibility factors are not mutually exclusive; as discussed in Section 3.5 heritable immune dysregulation and heritable neurodevelopmental vulnerability may co-occur in PANDAS-affected families [55,56]. A careful premorbid developmental and behavioural history—including temperamental anxiety, perfectionism, and early compulsive-like behaviours—is therefore an important component of the clinical assessment, both to estimate pre-test probability and to contextualise the significance of acute symptom change [5,17,56].

Families receiving a PANDAS/PANS evaluation may undergo repeated streptococcal serology, including the Cunningham Panel, which has not demonstrated adequate specificity in independent blinded evaluations [12,31]. Prolonged antibiotic prophylaxis and immunomodulatory interventions including IVIG and plasma exchange carry documented risks; evidence for their efficacy is limited to selected trial populations and requires diagnostic confidence that currently cannot rest on elevated streptococcal titres alone [16,57]. Antibiotic prophylaxis reduced exacerbations in one selected trial [58], and clinical benefit from immunotherapy has been reported in case series, but adequately powered randomised controlled trials with strict inclusion criteria are lacking [57,59,60]. The 2025 AAP clinical report acknowledged PANS as likely valid and recommended CBT and SSRIs as first-line treatment, while advising caution regarding invasive immunotherapies in the absence of confirmed autoimmune disease [16]. This guidance reflects the current state of evidence. A clinically relevant observation in this field concerns the differential psychological acceptability of competing aetiological frameworks for families and, sometimes, clinicians. A post-infectious explanation for tic onset—attributing new or worsening tics to a streptococcal infection—is often perceived as more immediately actionable and emotionally satisfying than a neurobiological or genetic model: it implies a discrete cause, a treatable trigger, and the possibility of prevention through antibiotic management. By contrast, accepting that a child’s tic disorder reflects a heritable neurodevelopmental trait—with strong familial aggregation, an intrinsic waxing–waning course, and limited immediate pharmacological reversibility—may be psychologically more difficult for families to integrate. This asymmetry in acceptability can inadvertently drive diagnostic confirmation bias, in which temporal co-occurrence of GAS exposure and tic exacerbation is readily interpreted as causal, while the neurodevelopmental hypothesis receives less weight despite stronger population-level epidemiological support. Clinicians should be aware of this dynamic and ensure that the communication of diagnostic uncertainty is balanced, is evidence-based, and does not inadvertently reinforce an aetiological attribution unsupported by available data. Appropriate psychoeducation about the natural history of primary tic disorders—including their typical onset in school-age children coinciding with the peak period of GAS exposure—is an essential component of the clinical encounter.

### 4.2. Research Priorities

Progress in this field requires prospective cohorts with predefined streptococcal infection documentation, standardised neuropsychiatric outcome measures, systematic family history collection, and pre-specified sample collection windows [9,10,17,52]. Retrospective referral cohorts are susceptible to recall bias and spectrum bias. The molecular biomarker work on anti-D1R and anti-D2R autoantibodies warrants prioritised investigation [27,28,29,30]. Validation in population-based or primary-care cohorts against diagnostically relevant comparators—including primary tic disorders, primary OCD, and healthy controls—using pre-specified blinded assessment is essential before clinical implementation. A validated accessible assay for these markers would substantially advance diagnostic practice. Therapeutic research requires adequately powered randomised controlled trials with strict inclusion criteria. Future studies should additionally address the potential role of pre-existing neurodevelopmental vulnerabilities and immunogenetic susceptibility factors [55,56]—including ASD, anxiety disorders, and familial autoimmune predisposition—as effect modifiers of neuropsychiatric presentation in the context of GAS infection, and should incorporate systematic sociodemographic characterisation of enrolled populations [15,19,25]. The ongoing NIH-supported prospective multicentre study of PANS will provide important epidemiological data [16].

### 4.3. Strengths and Limitations

This narrative review has several strengths. It integrates evidence across multiple domains—molecular biomarker biology, genetic epidemiology, clinical differential diagnosis and therapeutic guidance—providing a comprehensive and clinically oriented synthesis that complements and contextualises the 2025 AAP clinical report on PANS. The inclusion of large population-based cohort studies on the familial aggregation of tic disorders and of multi-cohort molecular studies on dopamine receptor autoantibody biology ensures that the review reflects the current state of the most methodologically robust evidence available. The explicit discussion of diagnostic pitfalls, including the risk of incidental temporal co-occurrence and the consequences of diagnostic imprecision, offers direct clinical utility beyond what is covered in existing guidelines.

This work has inherent limitations. As a narrative review, it is not a systematic review or meta-analysis; the literature search and article selection, while structured, were not governed by pre-specified eligibility criteria or independent duplicate screening, and are therefore subject to selection bias. The available evidence base derives predominantly from referral cohorts in high-income settings, limiting generalisability. Publication bias in favour of positive findings cannot be excluded. These limitations are consistent with the narrative review methodology and do not diminish the clinical relevance of the conclusions, but should be considered when interpreting the evidence synthesis presented.

## 5. Conclusions

PANDAS is a biologically plausible hypothesis supported by converging experimental evidence. The identification of anti-D1R autoantibodies as candidate neuropsychiatric biomarkers and anti-D2R as candidate movement biomarkers, and the characterisation of striatal cholinergic interneuron dysfunction as a candidate cellular mechanism represent genuine scientific advances warranting continued investigation [27,28,29,30]. Current evidence does not establish elevated ASO titres or anti-DNase B titres, or commercially available neuronal antibody panels as specific individual-level diagnostic markers for PANDAS. GAS infection, tic disorders, and OCD all occur commonly in overlapping paediatric age ranges, and prospective blinded studies have not demonstrated a consistent population-level association between GAS infections and neuropsychiatric exacerbations [9,10,51]. Primary tic disorders and OCD carry substantial familial aggregation, documented in population-based cohort studies [36,37,38,39]. Family history for tic disorders, OCD, and related neurodevelopmental conditions is accordingly a relevant clinical element in the pre-test probability assessment. Children with acute-onset neuropsychiatric symptoms and abnormal movements warrant systematic evaluation that considers the full range of differential diagnoses, including primary heritable tic disorders, Sydenham chorea, autoimmune encephalitis, and genetic or metabolic conditions, alongside the possibility of PANDAS/PANS. The presence of a pre-existing neurodevelopmental condition such as ASD does not preclude PANDAS but substantially raises the threshold for diagnostic attribution, requiring strict application of all five PANDAS criteria and rigorous exclusion of symptom fluctuation related to the underlying condition [16,19]. This approach is consistent with current guidance from the AAP [16] and with the available evidence base. A further clinically important consideration concerns the consequences of misdiagnosis in the absence of validated biomarkers: the inappropriate attribution of neuropsychiatric symptoms to a post-infectious autoimmune mechanism may lead to the use of aggressive therapies—including prolonged antibiotic prophylaxis, intravenous immunoglobulin (IVIG), plasma exchange, and corticosteroids—in children who do not have documented autoimmune disease. These treatments carry significant risks, including immunological, infectious, and metabolic adverse effects, and may delay access to interventions of established efficacy, such as cognitive behavioural therapy and SSRI pharmacotherapy [16,57]. Rigorous application of diagnostic criteria and systematic exclusion of alternative diagnoses therefore represent not only an epistemological imperative but a clinical necessity for the protection of paediatric patients.

## Figures and Tables

**Table 1:** Principal differential diagnosis of hyperkinetic movement disorders and acute neuropsychiatric symptoms in childhood.

Condition	Onset	Movement Type	Serology	Distinguishing Features
Primary tic disorder/Tourette syndrome	Gradual; childhood	Motor ± vocal tics	ASO normal	Waxing–waning natural history; family history frequently positive; no prospective population-level streptococcal association
Sydenham chorea	Subacute; post-strep	Generalised chorea	ASO ↑ or normal (late)	Carditis/arthritis; anti-D2R ↑; Jones criteria met
PANDAS	Abrupt; episodic	Subtle choreiform ± tics	ASO may be ↑	Diagnosis of exclusion; strict criteria required; documented GAS infection
Autoimmune encephalitis	Acute; progressive	Dyskinesias; dystonia	Anti-NMDAR and others	EEG/CSF/MRI abnormalities; multisystem involvement; immunotherapy-responsive
Genetic/metabolic disorder	Variable	Chorea; dystonia; tremor	Normal (specific panel)	Wilson disease; NKX2.1 mutations; PRRT2; ADCY5; juvenile Huntington; metabolic screen

ASO = anti-streptolysin O; GAS = Group A Streptococcus; PANDAS = Pediatric Autoimmune Neuropsychiatric Disorders Associated with Streptococcal infections; ↑ = elevated.

## Data Availability

No new data were created or analysed in this study. Data sharing is not applicable to this article.
